# Comparison of Percussion and Roentgenograms of Pericarditis

**Published:** 1918-11

**Authors:** 


**Comparison of Percussion and Roentgenograms of Pericarditis.** Morris and Bader, Arch. des Mal. du Couer, Feb., mention the dispute as to the accuracy of the former method, especially as to the limits of effusion detectable by percussion. They have experimented with 4 cadavers, the pericardium being injected with 250 c.c. at a time up to 1500 c.c., the usual maximum of distensibility. The first change in percussion was noted above, when 250 c.c. had been injected. Retro-sternal dullness was detected with 500 c.c. except that in one cadaver it failed with 750. An obtuse cardio-hepatic angle could not be obtained even with 1500 c.c. The liquid was first detected by the X-rays at 150 c.c. In the sitting posture, both retro-sternal dullness and the enlargement of the shadow disappeared by gravitation. Regarding the maximum quantity of effusion, they quote Verney as having obtained 4000 c.c. while Sibson gives the total capacity of the normal pericardium as 420-660.

